# Executive functions in everyday life in children born small for gestational age - a pilot study of pre-term to full-term children 3 years and younger

**DOI:** 10.1186/s12887-025-05564-1

**Published:** 2025-03-15

**Authors:** Ida Kongstad, Suzanne Petersson

**Affiliations:** 1 Örebro University Hospital, Örebro, Sweden; 2 Faculty of Health and Life Sciences, MEO, Linnaeus University, Kalmar, Region Kalmar County Sweden

**Keywords:** Small for gestational age, Preschool child, Executive function, Language development

## Abstract

**Background:**

Children born small for gestational age (SGA) have shown an increased risk of developing cognitive impairment and more difficulties regarding academic performance later in life. However, it is not known whether cognitive impairment can be detected in very young children. This study aimed to investigate whether children born SGA, with a birthweight of ≤ 3 SD, aged 2:6–3:0 years, showed impairments of executive functions in everyday life based on parental ratings, compared to children born appropriate for gestational age (AGA).

**Methods:**

Thirty children with birth week 33–41, 15 in each group, were included. The children in the groups were matched based on gender, age at testing (± 3 months), and parental educational level. Cognitive development was measured with the Bayley-III assessment. The BRIEF-P was used for parental ratings of the children’s executive functioning.

**Results:**

In terms of development a statistically significant difference between groups was shown regarding language ability, where the SGA group performed slightly worse compared to the AGA group (MD = -10.5 index points; 95% CI = -18.7–2.2; *t*(14) = -2.7; *p* = 0.02). No statistically significant differences were found between groups regarding parental ratings on the BRIEF-P.

**Conclusions:**

The study found no significant differences in EF between children born SGA and AGA based on parental ratings. Given the small sample the lower language ability in the SGA group suggests potential EF impairments, which could be detected at a younger age than is presently customary. These findings underscore the need for further research using varied assessment methods and larger samples to better understand EF development in this population. Early discovery of EF impairment is important for enabling adequate interventions for family, school, and health care.

## Background

About 5% of newborn children weigh ≤ 2 SD less than expected [1], and are thus defined as small for gestational age, SGA [2]. Growth retardation during fetal life has a variety of causes, such as low pregnancy weight, preeclampsia, infections (e.g. toxoplasmosis, rubella), smoking or drug abuse during pregnancy, placental factors (e.g. placenta previa, low-lying placenta), or genetic disorders [3].

Previous studies have shown that children born SGA have an increased risk of cognitive impairments and more difficulties regarding academic performance later in life compared to children born appropriate for gestational age, AGA [4–7]. Several studies have, in addition to general cognitive function, also studied executive functions (EF) in children born SGA using psychological skill tests [8–12]. The concept of EF involves a number of basic neuropsychological functions that enable initiation, planning, and execution of tasks and/or affect monitoring [13]. Unlike other cognitive functions, studies on EF are about *if* and *how* a person solves problems, rather than *what* or *how much* a person can perform. Impaired EF has a global impact on behaviour and can directly affect cognitive functioning [14]. Impairment of EF in early childhood has been shown to correlate with more difficulties regarding academic performance later in life. Mulder et al. [15] e.g., showed that EF at age two was a strong predictor of mathematical and reading ability at age five. EF also has a general impact on language development [16].

Most studies using psychological ability tests have shown more executive difficulties among individuals born SGA compared to those born AGA. This finding was consistent across evaluations of school-aged children, young adults, and adults [8–10, 12, 17, 18]. On the other hand, in a few studies the results have been contradictory, and no statistically significant differences between groups have been found [19, 20]. Although psychological performance-based tasks of EF purport to examine core abilities of EF (working memory, cognitive speed, impulse control, inhibition), it has been questioned whether this truly reflects EF in everyday life [21]. The tests are usually presented in a stripped-down and structured environment, with abundant support and minimal distractions. Reality is rarely like that, thus, it can be difficult to say whether performance-based tasks really reflect the abilities required in everyday life [21].

A few studies have chosen to examine EF in everyday life using validated rating scales, e.g., the Behavior Rating Inventory of Executive Function, BRIEF [22, 23]. Previous studies that aimed to examine EF in everyday life using the BRIEF found no significant differences between children born SGA and children born AGA at age 7–8 [11, 18]. On the other hand, parents of adult children born SGA reported greater difficulties in all BRIEF areas compared to parents of adult children born AGA [24]. Adults born SGA, who completed the self-report version of the BRIEF, however, reported less executive difficulties than their parents, and also fewer difficulties compared to adults born AGA [24].

It is difficult to draw conclusions from previous studies of EF because, regardless of whether performance-based tests or rating scales were used, there are differences regarding study design, group selection, and confounding factors, not least concerning the few studies on cognitive development and behaviour in children ≤ 6 years born SGA [25]. To our knowledge, there is currently only one published study of EF for children 3–6 years old born SGA [26]. This study focused mainly on ADHD symptoms, but when using Conner’s rating-scale the children born SGA showed statistically significant higher ratings of attention deficit than the children of the same age born AGA.

Thus, the level of evidence for studying EF in children born SGA before the age of 6 is inadequate. As with other cognitive functions, such as language, EF can be measured over time even at early ages. The fact that studies on young children’s EF are scarce, may to some extent, depend on limited assessment methods [27]. Another reason may be that EF develops and changes considerably during the preschool age [15, 28]. It is likely that the structure of EF in preschool children is different compared to older children and adults [29], thus adapted assessment methods are required for this group. Although studies investigating EF in everyday life have, in general, shown no statistically significant differences between children born SGA and AGA, there is still reason to believe that such may exist. Among other things, most studies on EF that have used performance-based tasks have shown differences. It has been suggested that if executive difficulties are identified early interventions can be implemented, and thus reduce the negative impact on other developmental and cognitive abilities [14, 28]. Since EF has a general impact on cognitive development this may be a contributing cause of differences between children born SGA and AGA regarding academic success. Thus, there remains a need to investigate whether executive difficulties in everyday life occur in children born SGA, and if they are detectable in children aged 6 and under.

### Aim

The aim of this pilot study was to investigate whether children born SGA, with a birthweight of ≤ 3 SD, aged 2:6–3:0 years, demonstrate greater impairments in executive functions in everyday life based on parental ratings, compared to children born AGA. Our hypothesis was that children born SGA would exhibit more pronounced EF impairments in daily life when compared to age and gender-matched children born AGA.

## Methods

### Participants and procedure

**Fig. 1:**
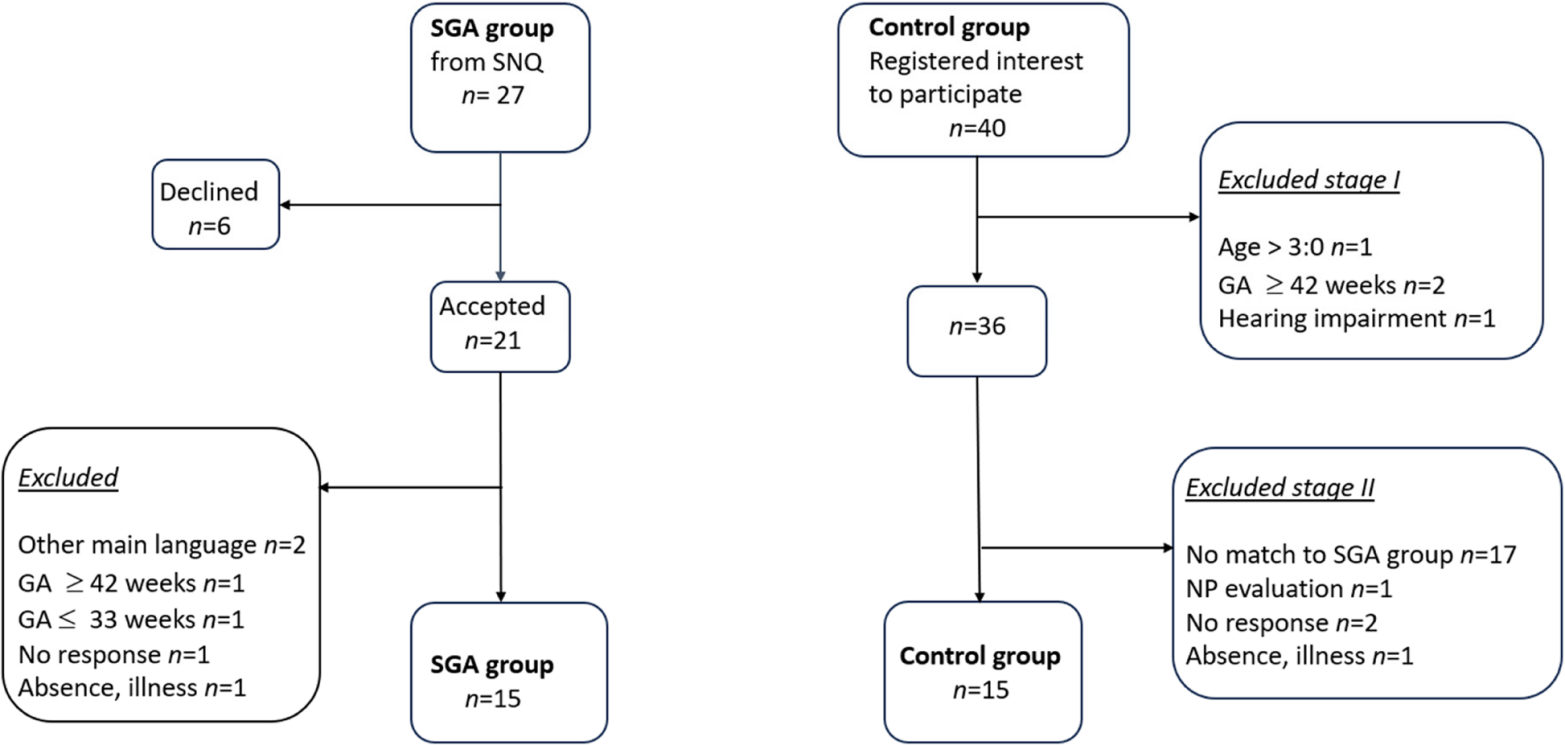
Flowchart of the inclusion process. GA = Gestational age, NP = Neuropsychiatric, SGA = Small for gestational age, SNQ = the Swedish Neonatal Quality register

During 2019–2023 possible participants were identified through the Swedish Neonatal Quality register (SNQ) within four Health Catchment Areas (HCA) in southern and central Sweden. All children within the study population were included in the national follow-up program for neonatal at-risk children [30]. Children with a gestational age of < 28 weeks, a birth weight below −3 SD, morphological brain injuries, significant asphyxia, severe encephalopathy due to various etiologies, cerebral infections, or other severe illnesses were included in the national follow-up program for neonatal at-risk children. Data extraction from the SNQ was administrated by the chief medical secretary at each participating paediatric clinic (Fig. 1). Children aged 2:6–3:0 years, born SGA with a birth weight ≤ 3 SD, were included. As the participants were part of the national follow-up program, it was not possible to include or identify children with milder SGA (birth weight between −2 and −3 SD). To ensure broader inclusion, children born moderate-to-late preterm and those born at term (gestational age 33–41 weeks) were included. Moderate-to-late preterm children typically exhibit only minor cognitive and learning impairments compared to term-born peers [31]. Extremely preterm children, who are at higher risk of neurodevelopmental delays [32], as well as very preterm children, were excluded to minimize age disparities between the study and control groups.

Families with children who met the inclusion criteria received a letter with information about the study and an invitation to participate. Children with confirmed developmental abnormalities, visual or hearing impairment, were excluded from the study, as were children without a parent whose main language was Swedish. Of 27 identified families, six families declined participation. Four families failed to meet the inclusion criteria, and two families dropped out before the initial meeting. A total of 15 families completed the study. The participants were matched with 15 voluntary controls born AGA, recruited using an information letter distributed at child health care units and preschools. Controls were matched by gender, age at testing, and both parents’ educational levels. A total of 40 families registered their interest to participate, of which four did not meet the inclusion criteria. Of the remaining 36 families, 21 were rejected, as they either did not match the study participants (*n* = 17), needed further neuropsychological impairment evaluation (*n* = 1), or were ill at the time of the test (*n* = 1). The remaining 15 families completed the study (Fig. 1). The mean gestational week for the children in the SGA group was *M* = 37 weeks and 1 day (range: 33 weeks and 2 days to 41 weeks and 6 days). The mean birthweight in this group was *M* = 1890 g (1225–2476 g).

Of the 30 children included 10 were girls and 20 were boys. All children were matched regarding test age ± 3 months, except for two pairs with an age difference of 4 and 5 months, respectively. The mean gestational week for the children in the AGA group was *M* = 39 weeks and 7 days (range: 35 weeks and 4 days to 41 weeks and 4 days). The mean birthweight in this group was *M* = 3559 g (2334–4410 g). Basic demographic data for participating families are presented in Table 1.

**Table 1:** Gender, test age, and parents’ educational levels

	**SGA (*n* = 15)**	**AGA (*n* = 15)**
-Boy	10	10
-Girl	5	5
-Age at test *M* (range)	33.0 (30–35)	34.2 (30–36)
Parent E1		
-Elementary School	0	0
-Senior High School	5	4
-University	10	11
Parent E2		
-Elementary School	2	2
-Senior High School	10	9
-University	3	4

Participants in the control group were matched by gender, age at testing, and both parents’ educational levels

Number of the participating children, their gender, and test age in months

E1 = Parent with the highest level of education in the couple. E2 = Parent with the lowest/equivalent level of education in the couple

*M* Mean*, SGA* small for gestational age, *AGA* appropriate for gestational age

To assess the level of cognitive development, the Bayley-III developmental battery [33] was used. Testing was performed in a clinical setting by the same psychologist. Since the psychologist also served as the study coordinator, the study was not blinded. Before the visit the parents had scored the BRIEF-P [22] to assess the level of EF in daily life. The parents were offered a follow-up meeting to discuss the results. If clinical deviations from the norm were observed the families were referred to the proper authority.

#### Ethical approval and consent to participate

The study was conducted according to the principles of the Helsinki declaration. Prior to the tests and questionnaires, the legal guardians of the participants involved in this study were provided with written and oral information about the study. Informed consent was obtained. They were informed that participation was voluntary, and that participation or refusal would not affect future treatment. The legal guardians of the participants in the study were informed that presentation of the data would be handled with confidentiality so that no data could be traced to any single informant. Written consent to participate in the study was provided. The study was approved by the Swedish Ethical Review Board (No 2019–0238 and No 2022–03090-02).

### Measures

#### Behavior Rating Inventory of Executive Function: Preschool version (BRIEF-P)

The BRIEF-P is a standardised questionnaire for the assessment of EF in children aged 2–5:11 years [22, 23]. The questionnaire can be answered by people who are in close contact with the child, e.g., parents or preschool teachers. The questions are based on observed behaviour in everyday situations, providing a picture of the child’s EF in real-life environments [22, 23]. The BRIEF-P consists of 63 statements within five scales that deal with different aspects of EF: *Inhibit*, *Shift*, *Emotional Control*, *Working Memory*, and *Plan/Organise*. The scales can be combined into three broader indexes: Inhibitory Self-Control Index, Flexibility Index, and Emergent Metacognition. In addition, there is a superordinate index – the Global Executive Composite (GEC). There are also two validity scales: for inconsistence and negative response patterns [22, 23]. The BRIEF-P has shown satisfactory psychometric properties regarding internal consistency (*α* = 0.85 to 0.95 for parental ratings), interrater agreement, and test–retest stability (*r* = 0.78 to 0.90 for parent ratings) [22, 23]. Raw scores are converted into T-scores *(M* = 50, *SD* = 10) to provide information about the child’s results in relation to children of the same age. A T-score ≥ 65 is considered clinically significant, i.e. a higher T-score means more difficulties [22, 23].

#### Bayley scales of infant and toddler development-III (Bayley-III)

The Bayley-III [33] is an individually administered test battery for children aged 1–42 months. It contains five scales: *Cognitive*, *Language* (receptive and expressive communication), *Motor* (gross and fine motor skills), *Social-Emotional*, and *Adaptive Behavior Scales*. The present study used the first three scales which have shown satisfactory psychometric properties regarding internal consistency (*α* = 0.94 to 0.98), interrater-agreement, and test–retest stability (*r* = 0.80 to 0.87) [33, 34]. The raw score in the Bayley-III is converted to an index score, which enables comparison of the child’s performance in relation to children of the same age group *(*M = 100, SD = 15). A higher index score means a better result. The Bayley-III is adapted to and translated into Swedish, but since there is no Swedish norm data the interpretation was based on American norm data [33].

### Statistical analyses

All statistical analyses were performed in R, version 4.3.2 [35]. Correlations between Bayley-III and BRIEF-P scorings were computed using Pearson’s *r*. Differences in cognitive, language, and motor development, as well as scores in the BRIEF-P, were analysed with paired *t*-tests.

## Results

**Fig. 2:**
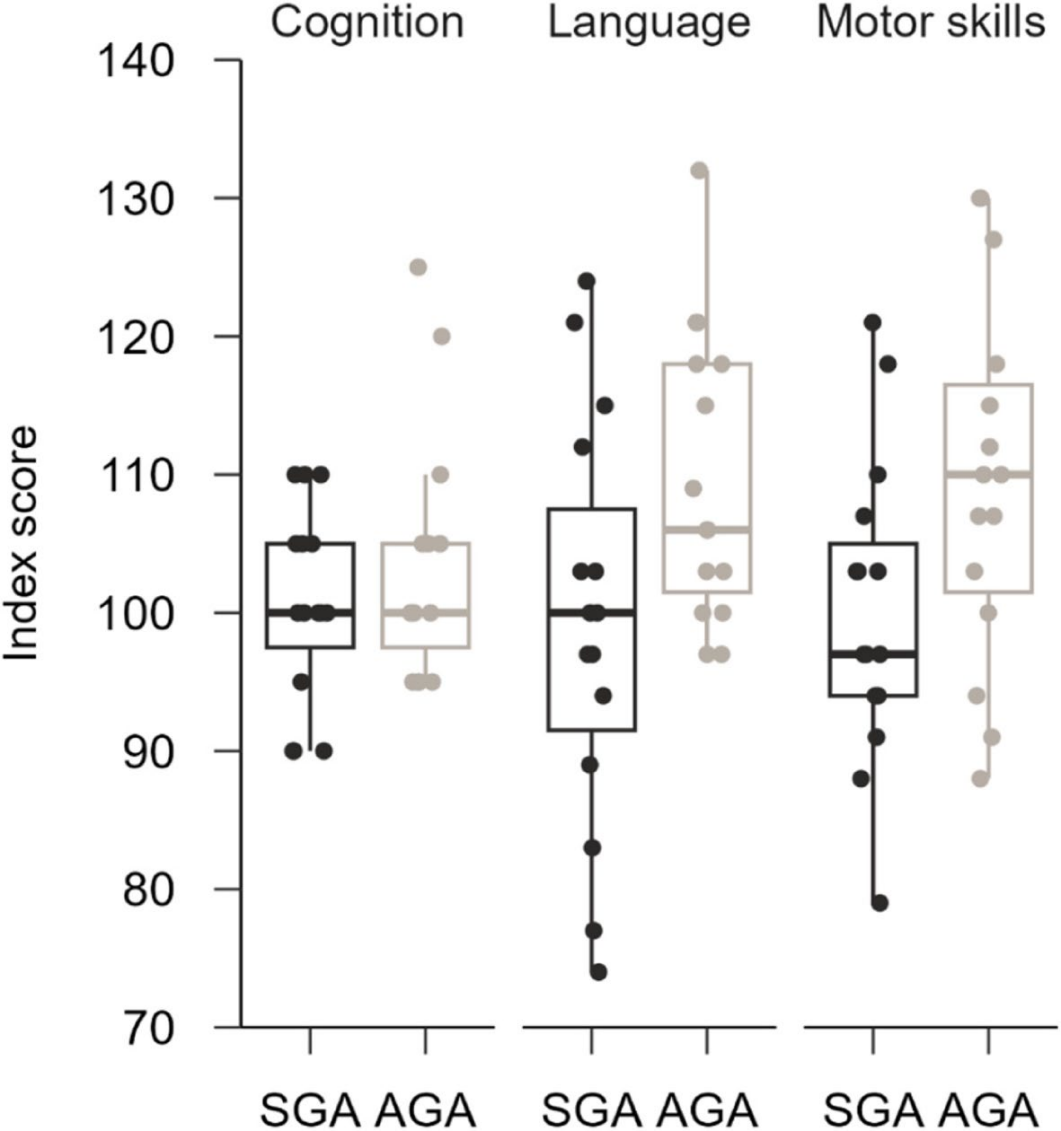
Bayley-III index points. Higher scores indicate better results

Only two of the four HCAs in the study had children who met the criteria of the study reported in the SNQ. The results on Bayley-III are illustrated in Fig. 2 and Table 2. The parents of the children in the SGA group provided the ratings as follows, compared parent ratings for the AGA group: *Cognitive* scale (*MD* = −2.7 index points; 95% *CI* = −9.4–4.0; *t*(14) = −0.85; *p* = 0.41), on the *Language* scale (*MD* = −10.5 index points; 95% *CI* = −18.7–2.2; *t*(14) = −2.7; *p* = 0.02), and on the *Motor* scale (*MD* = −9.3 index points; 95% *CI* = −19.7–1.0; *t*(14) = −1.9; *p* = 0.07). The differences were small, the variation high, and the only statistically significant difference (*p* < 0.05) was shown for language ability.

**Table 2:** Bayley-III and BRIEF-P scorings

	**SGA *M* (*SD*)**		**AGA *M* (*SD*)**		
		Range		Range	*P*
**Bayley-III**					
Cognition	101 (7.04)	90–110	103 (9.00)	95–125	0.407
Language	99.3 (14.8)	74–124	110 (10.5)	97–132	0.017
Motor skills	100 (11.0)	79–121	109 (13.2)	88–130	0.073
**BRIEF-P**					
Inhibit	43.8 (8.29)	34–62	46.8 (7.71)	37–62	0.304
Shift	43.1 (6.06)	38–55	43.6 (5.55)	37–58	0.844
Emotional control	44.1 (10.2)	35–66	48.7 (8.59)	38–72	0.222
Working memory	50.1 (10.8)	38–66	49.0 (8.01)	36–64	0.762
Plan/organize	44.5 (11.1)	32–66	44.7 (7.69)	32–58	0.939
GEC	43.6 (9.58)	32–59	46.4 (8.38)	34–65	0.421

Paired t-tests of the Bayley-III and BRIF-P scales, 95% Confidence interval

*AGA* Appropriate for gestational age (control group), *n* = 15, *GEC* Global Executive Composite, *M* Mean, *SD* Standard deviation, *SGA* Small for gestational age, *n* = 15

**Fig. 3:**
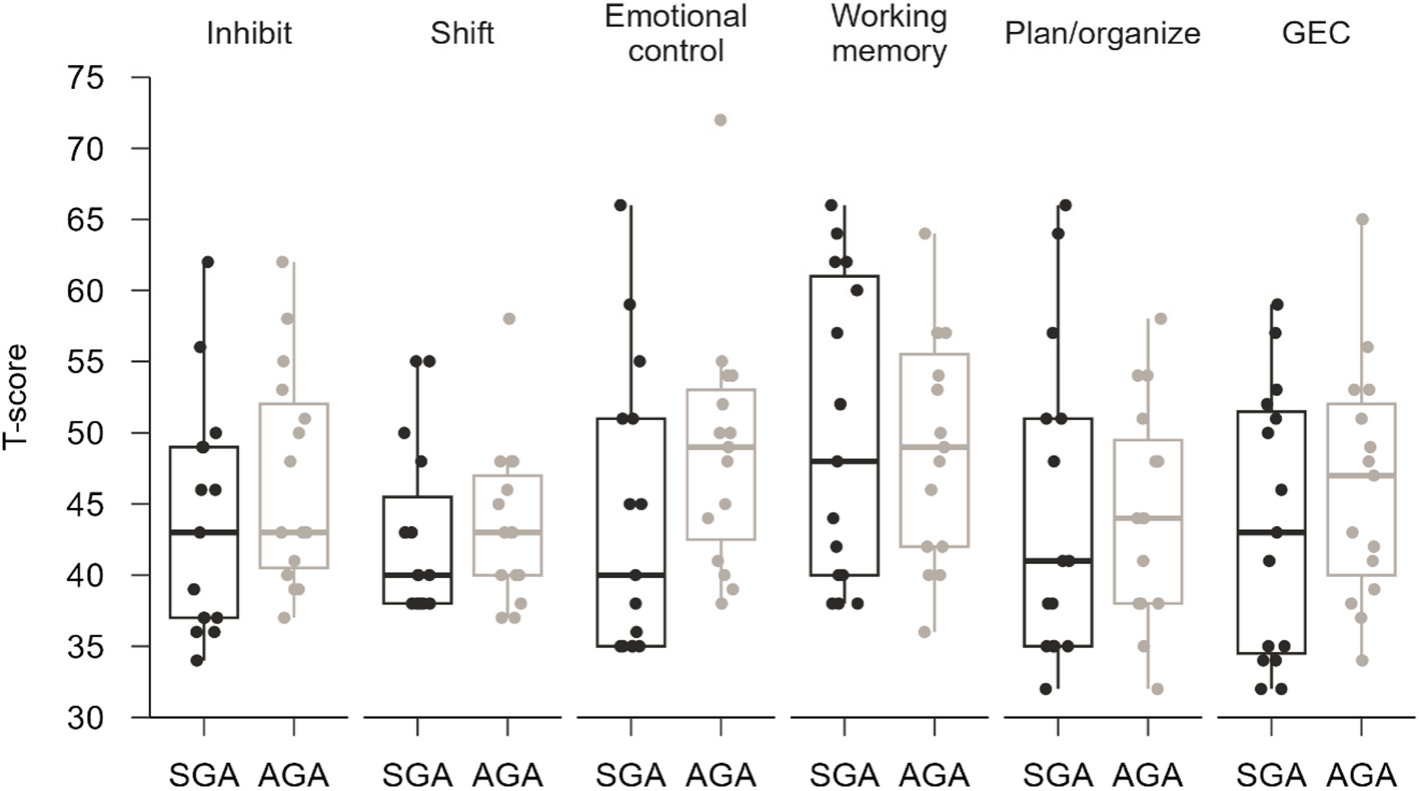
BRIEF-P T-scores. Higher T-scores indicate worse results

The results from the BRIEF-P are illustrated in Fig. 3 and Table 2. Two children in the study group were estimated to have a T-score ≥ 65 within one or more of the scales measuring *Emotional Control*, *Working Memory*, and *Plan/Organise*, while one child in the control group was estimated to have a T-score ≥ 65 within *Emotional Control*. This child (in the control group) also had an elevated score on the *General Executive Composite index* (*GEC*). The parents of the children in the SGA group provided the ratings as follows, compared parent ratings for the AGA group: *Inhibition* (*MD* =—3 T-scores; 95% *CI* = −9.0–3.0; *t*(14) = −1.07; *p* = 0.30), *Flexibility* (*MD* = −0.5 T-scores, 95% *CI* = −5.4–4.5; *t*(14) = −0.2, *p* = 0.84), *Emotional Control* (*MD* = −4.7 T-scores, 95% *CI* = −12.5–3.2; *t*(14) = −1.3, *p* = 0.22), *Plan/Organize* (*MD* = −0.3 T-scores; 95% *CI* = −7.6–7.0; *t*(14) = −0.1; *p* = 0.94), and *GEF* (*MD* = −2.8 T-scores; 95% *CI* = −10.0–4.4; *t*(14) = −0.8; *p* = 0.42). The differences were small, the variation was high, and no statistically significant differences were found.

**Fig. 4:**
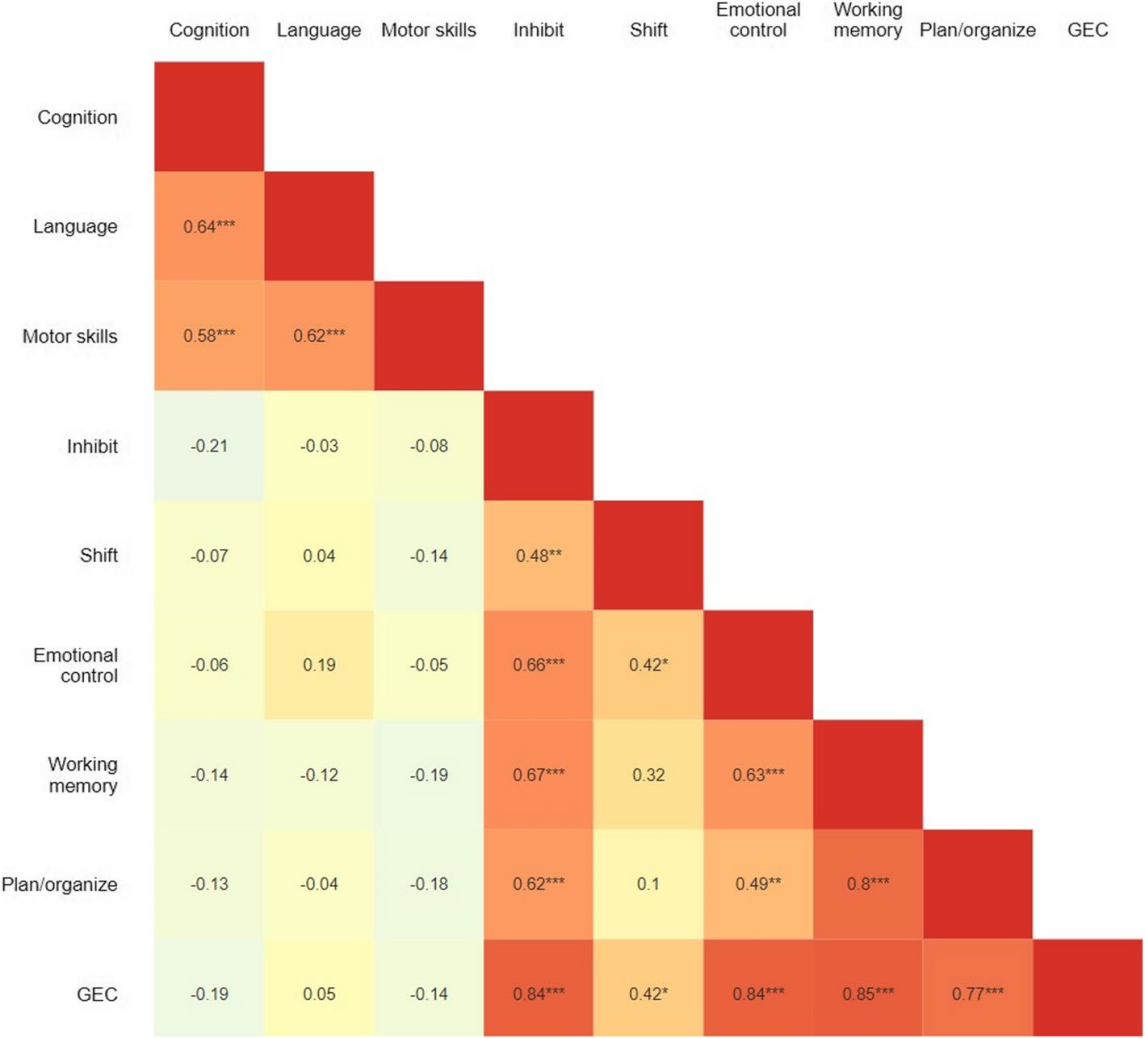
Correlation matrix between Bayley-III and BRIEF-P scales. Correlations between the Bayley-III subscales: *Cognitive*, *Language*, and *Motor skills*, and the BRIEF-P scales: *Inhibit*, *Shift*, *Emotional control*, *Working memory*, *Plan/organise*, and the BRIEF-P *Global Executive Composite* (*GEC*). Correlations between the scales were computed using Pearson’s r. *p < 0.05, **p < 0.010, and ***p < 0.001

Figure 4 displays the correlations between the Bayley-III scales and the BRIEF-P scales, ranging from non- significant *r* = −0.03 (Bayley-III *Language* and BRIEF-P *Inhibit*) to a significant and high correlation, *r* = 0.8 (BRIEF-P *Working memory* and *Plan/organise*). The three Bayley-III scales showed statistically significant correlations (*p* < 0.001) within the range of *r* = 0.58–0.64, while the correlations between the Bayley-III scales varied from non-significant to highly significant (*r* = −0.05 to *r* = 0.80). The Bayley-III GEC scale and all its subscales, except one, were strongly and significantly correlated (*r* = 0.77- *r* = 0.85, *p* < 0.001), with the weakest correlation observed between the GEC and *Shift* subscales (*r* = 0.42, *p* < 0.05).

## Discussion

The present study aimed to investigate whether children born SGA showed greater impairments in EF in everyday life compared to children born AGA. This study sought to enhance the understanding of EF in young children born SGA, with the goal of improving opportunities for early detection and targeted support. The purpose of early detection is to enable proactive interventions before these impairments contribute to deficits in language development, school performance, and, ultimately, psychological well-being. Previous studies have mainly investigated older children or used other methods [11, 18]. The complexity of finding reliable methods of testing, and enough participants, may be a reason for the lack of scientific evidence in the field. Until there is reliable information it is difficult to know whether, for example, if it is right to exclude these children from structured follow-up programs. The fact that interventions differ between HCAs is also a problem since the families are not offered equal care.

The current pilot study investigated EF in preschool children born SGA compared to children born AGA, using the Bayley-III and BRIEF-P assessments. Our hypothesis was that children born SGA would show greater impairments of EF in daily life compared to age and gender-matched children born AGA. No statistically significant differences in EF were found based on parent ratings. However, the children born SGA showed slightly lower T-scores on the BRIEF-P, except for *Working Memory,* where they were rated higher. In general, the differences were small and not statistically significant, and the variation was large, especially in the SGA group. Thus, the results did not support our hypothesis that preschool children born SGA would show greater impairments regarding EF in everyday life compared to children born AGA. Despite this, the SGA group demonstrated statistically significant more difficulties on the Bayley-III *Language* scale, which could potentially indicate executive impairments, since language and EF have a reciprocal relationship [16].

Studies of EF in children aged < 6 years born SGA assessed with questionnaires are few. Shariat et al. [26] published a study with a similar aim to the current study but contrary to our results, it showed statistically significant differences between children born SGA and AGA regarding pre-school teachers’ ratings on the Conner’s Teacher Rating Scale. In this study the children born SGA showed more difficulties with attention compared to the children born AGA. That study included children in the age range of 3–6 years, but the age distribution was not reported. Since much happens with children’s executive development during the first years of life [28] it is difficult to transfer these results to the present study. The studies also differ in their selection of respondents, which may further contribute to the varying outcomes.

Most studies that have primarily used questionnaires as assessment of EF have studied children of school age and older. In two studies by Tanis et al. [11, 18] parents of children born SGA completed the BRIEF-P but, in line with our results, no statistically significant differences between children born extremely premature, moderately premature, or at full term, were shown. Heinonen et al. [24] had young adults (21–30 years old) born SGA, their parents, and a control group to complete the BRIEF, and found that the parents of young adults born SGA reported more EF impairments in their children compared to parents in the control group. Parents to participants in the SGA group also reported more EF impairments than their children reported about themselves. However, in this study, the BRIEF ratings were combined with EF tests (The Trail-making test, the Stroop Test, and verbal fluency), which correlated more to the parent’s reports than their own [24]. Heinonen et al.’s [24] study highlights potential issues with self-rating scales since they are based on the participant’s subjective experiences. In studies using skill tests for evaluating EF the majority have demonstrated more impairments in groups of persons born SGA [8–12]. Thus, there is reason to assume that children born SGA as a group do have more EF impairments despite the results of this study.

### Limitations

The matching of groups on gender, age, and parental education level strengthens this study, although matching by birth week could have added even greater strength. There are, however, limitations: overall, the differences between the groups were small and variations within the groups were large. The study’s small sample size poses a risk of type 2 errors, affecting the robustness of the conclusions. Another limitation is the sampling method, which exclusively included children in the national follow-up program for neonatal at-risk children, thereby excluding those with birth weights between −2 and −3 SD. Data extraction from the SNQ was carried out by the chief medical secretary at each participating paediatric clinic, suggesting that more participants might have been included if data collection had been conducted directly via the SNQ. Furthermore, such an approach would have yielded detailed information on the initially excluded children—information currently unavailable due to the selection process being managed by secretaries. This information could have contributed to a more comprehensive understanding of the SGA group.

When the study began a larger sample of children meeting the inclusion criteria was available, and enrolment targeted only one HCA. However, during the study these conditions changed, and the HCA – like many other HCAs in the country – chose to exclude children born SGA (−3 SD) from the neonatal at-risk follow-up due to lack of resources. This resulted in that recruitment could no longer be made through the register from this HCA, thus three more HCAs were involved. Eventually, only one of the added HCAs was able to identify and contribute participants to the study.

It is possible that impairments in EF may not yet be sufficiently discernible in the studied groups. One could speculate whether the outcome of our study would have been different if we had included BRIEF ratings from preschool staff, who are less emotionally involved observers and have a broader experience of child development. There is a risk of response bias since parents might underreport their child’s difficulties due to emotional involvement or sensitivity. Additionally, parents’ limited experience with child development, often due to having few or no other children for comparison within the home environment, may further contribute to this bias.

The fact that the parents in the SGA group scored better EF in everyday life for their children compared to parents in the AGA group contradicts previous research on the subject, but could support that kind of response bias. Voluntary participation might have skewed the sample towards more concerned parents (especially in the control group). It may also be difficult for parents, regardless of whether they have one or more children, to know when an ability or a behaviour deviates from average development. Preschool teachers would play an important role in identifying these children due to their experiences of large groups of children. Thus, there is a need for further studies with preschool teachers’ ratings of the children’s EF.

Using the Bayley-III to assess developmental levels in children can also be problematic. It is known that the Bayley-III overestimates the index scores in relation to the child’s actual abilities within all sub-scales [36]. Additionally, Bayley-III test scores have shown to be unstable over time and should instead be considered as current descriptions of children’s skills [37]. The current Bayley-III index scores should thus be interpreted with this in mind and may fail to correctly identify the presence of developmental deviations. The current study is, however, comparative and any methodological errors are thus similar for both groups. Thus, comparisons between groups should not be affected. The difficulty in finding reliable measurement methods and a sufficient number of participants may explain the lack of scientific evidence in this field.

## Conclusions

The present study indicates that EF difficulties in children born SGA can be identified earlier. This finding is important because these children are at an increased risk for impairments in executive and cognitive functions, particularly in language development. Additionally, the study underscores the challenges in detecting EF difficulties in young children through parent-scoring questionnaires and emphasises the importance of incorporating observations from preschool teachers to mitigate response bias and provide a more objective assessment of EF in young children.

Without reliable data, it is challenging to determine, for instance, whether excluding these children from structured follow-up programs is appropriate. Additionally, the variability in interventions across HCAs highlights a gap in standardised care, calling for further research and consistent follow-up programs to ensure equal and proactive support for children born SGA.

## Data Availability

The datasets used and/or analysed during the current study are available from the corresponding author on reasonable request.

## Abbreviations

ADHD: Attention-Deficit/Hyperactivity Disorder
AGA: Appropriate for Gestational Age
BRIEF-P: Behavior Rating Inventory of Executive Function- Preschool Version
EF: Executive Function-/s/-ing
GEF: General Executive Composite index
HCA: Health Catchment Area
SD: Standard Deviation
SGA: Small for Gestational Age
SNQ: The Swedish Neonatal Quality register
